# Effects of housing conditions on stress, depressive like behavior and sensory-motor performances of C57BL/6 mice

**DOI:** 10.1186/s42826-024-00193-8

**Published:** 2024-02-18

**Authors:** İsmail Abidin, Hatice Keser, Elif Şahin, Hilal Öztürk, Harun Başoğlu, Ahmet Alver, Selcen Aydin-Abidin

**Affiliations:** 1 Faculty of Medicine, Department of Biophysics, Karadeniz Technical University, Trabzon, Turkey; 2 Ataturk Vocational School of Health Services, Afyonkarahisar University of Health Sciences, Afyonkarahisar, Turkey; 3 Faculty of Medicine, Department of Biochemistry, Karadeniz Technical University, Trabzon, Turkey

**Keywords:** Individually ventilated cages, Housing, Stress, Sensory-motor function, Depression, Mouse

## Abstract

**Background:**

The effects of housing conditions on animal physiology, behavior or stress are still debated. The aim of this study was to investigate the effects of three different housing systems, individually ventilated cages (IVC), classical small cages with floor surface area of 500 cm^2^ (CC500) and classical large cages with floor surface area of 800 cm^2^ (CC800) on body weight, sensory-motor performances, depression-like behavior, plasma corticosterone and brain oxidative stress parameters in C57BL/6 mice. The mice housed in one of the cages from birth to 6 months of age. Hang wire and adhesive removal tests were performed to evaluate somatosensory and motor performances. The extent of depression was determined by the forced swim test. Blood corticosterone levels were measured. In addition, brain malondialdehyde (MDA), total antioxidant status (TAS) and total oxidant status (TOS) levels were analyzed.

**Results:**

The depression-like behavior of the groups was similar. Although there were no significant differences in hang wire test among groups, CC500 group required longer durations in adhesive removal test. The body weight and plasma corticosterone levels of CC800 group were significantly higher than other groups. The oxidative stress parameters were highest in CC500 cage.

**Conclusions:**

Our study showed that the least stressful housing condition was IVC cage systems. Interestingly, the number of mice in the classical cages had a significant effect on stress levels and sensory-motor performance.

**Supplementary Information:**

The online version contains supplementary material available at 10.1186/s42826-024-00193-8.

## Background

Many types of housing systems are used for the rearing and housing of laboratory animals in different laboratories or facilities. Animal facilities generally use traditional classical cages (CC) and more recently individually ventilated cage (IVC). Development of the IVC system began in 1963 at the Jackson Laboratories (Bar Harbour, ME04609, USA) and, current version of the IVC system has been used successfully since 1981 [1, 2]. The use of IVC systems has several advantages: more animals can be housed in limited space, animals are protected from unwanted outside influences and organisms, and personnel working with the animals are protected from risks such as allergens from animals. Clough et al. [3], have shown that the use of IVC systems significantly reduces animal aeroallergens in the room, as well as the transfer of airborne particles from the room into the cages [3]. There are conflicting reports as to whether or not IVC systems induce stress. IVC systems have been shown to protect mice from stress by isolating the odors and sounds of others. This limited sensory input from the outside can possibly be considered as isolation [4]. In contrast to these results, animals reared in IVC systems have been shown to be more stressed than other mice in classical cages. The air exchange rates in IVC systems are quite high. For this reason, IVC systems are thought to induce chronic cold stress in mice by assessing non-shivering thermogenesis and brown adipose vacuolation [5]. In addition, rodents housed in IVC cages showed increased c-Fos expression in the paraventricular nucleus of the hypothalamus [6], which is an indicator of stress responses [7]. Additionally, animals in most IVC systems, have little opportunity to climb [8], and cage rack vibrations can be stronger in IVC systems [9]. It is a fact that the use of IVC systems in research laboratories is increasing. Different findings from different laboratories resulting from the same experiment with the same species are often attributed to differences in environmental or methodology. Therefore, it is important to evaluate the effects of IVC systems on animal behavioral.

The effects of IVC systems on animal behavior have been reported. Logge and colleagues [10] investigated the behavioral performances of mice reared in IVC systems and found that IVC systems had no significant effects on cognition, habituation, exploration and locomotion whereas IVC had anxiety-like effects in the elevated plus maze. In addition, mice housed in IVC, showed more social interaction with other mice unfamiliar to them [10]. Åhlgren and Voikar [11], found that anxiety-like behavior increased in IVC system but they observed no difference in social interaction, locomotor activity, and immobility time in the forced swim test [11]. Moreover, the IVC systems have been shown to modulate brain neurotransmitter systems particularly the serotonin (5HT) and dopamine (DA) turnover [12, 13]. It has been reported that the anxiety behavior of mice is influenced by IVC systems. While a motor free ventilated system reduced the anxiety, a motor driven IVC system did not exacerbate anxiety behavior compared to an open top cage [14]. However, another study showed that IVC cage systems can have different effect on anxiety like behavior and that certain IVC cages induce anxiety on mice [15]. As can be seen from the studies, the findings on the IVC systems are not consistent. In the present study, we aimed to investigate the effects of 3 different cage systems. We compared.

i. the effects of IVC cages and classical cages as well as
ii. classical cages with different number of mice living in.

The first caging system was the IVC system (310 × 160x130 mm, floor surface ≈500 cm^2^, Plexx), the second cage system was a type 2L open top classical cage (floor surface ≈ 500 cm^2^, 310 × 160x135 mm, Tecniplast) (CC500) and the third cage system was a type 3 open top classical cage (floor surface ≈800 cm^2^, 370 × 210x180 mm, Tecniplast) (CC800). To our knowledge this is the first study to systematically investigate the effects of IVC systems on body weights, depressive behavior, sensory-motor performances, and oxidative stress parameters of cortex and hippocampus. Furthermore, the mice in this study, lived in the same cages from birth to 6 months of age.

## Methods

### Animals and husbandry

**Fig. 1:**
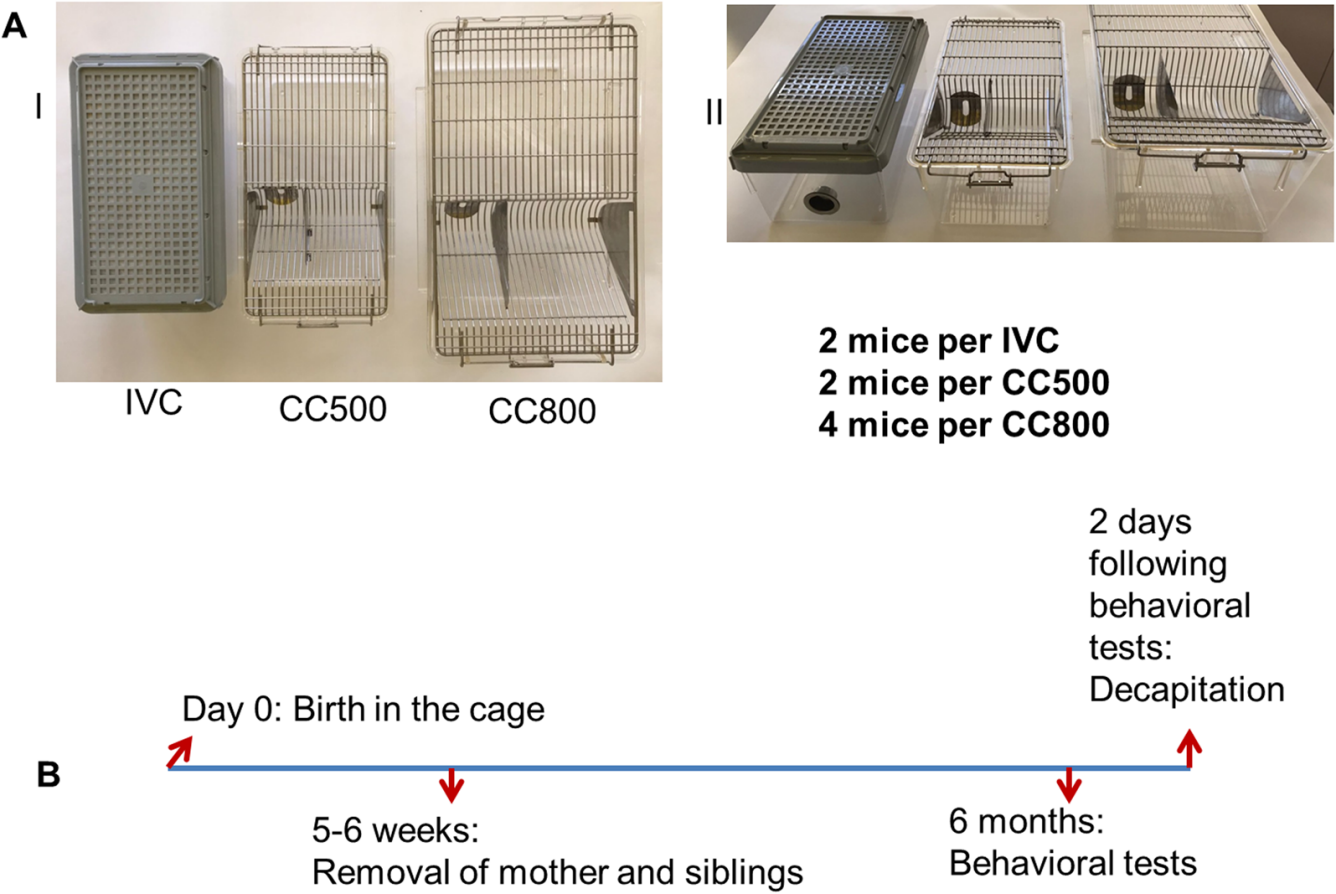
The cage types used and the time line of this study. **A **I Overhead and II Side view pictures of the cages. IVC (individually ventilated cage), CC500 (classical open cage with a floor area of 500 cm^2^) and CC800 (classical open cage with a floor area of 800 cm^2^) are commonly used cage types for mice housing. **B** The time line of the study is shown. The mice born in a particular cage type remain in the same cage throughout its life time. The siblings and parents were removed after 5–6 weeks. At the age of 6 months, the behavioral tests were performed, 1 day after which the mice were sacrificed

This study was approved by Karadeniz Technical University Animal Care and Ethics Committee. The approval number was 2020–152. The mice used in the study were provided by Karadeniz Technical University Surgical Application and Research Center and all experimental procedures were performed at this center. Experimental procedures on animals were conducted in accordance with the European Convention for the Protection of Vertebrate Animals. A total of 30 male C57BL/6 J strain (JAX Jackson Laboratories) mice were divided into three groups as IVC, CC500 and CC800. CC500 refers to open top classical cages with a floor area ≈ 500 cm^2^ and CC800 refers to open top classical cages with floor area ≈ 800 cm^2^. IVC cage system was PLEXX Rair IsoSystem Ventilated racks and the cages were WorldCage500 Micro-Isolator (Nedherlands). The floor area of an IVC cage was also ≈ 500 cm^2^ which is comparable to the CC500. Examples of each type of cage used were shown in the Fig. 1. The animal facility was maintained at 23 ± 1 °C with 55% humidity and a 12-h/12-h light/dark cycle (lights on from 7:30 a.m. to 7:30 p.m.). All the animals were fed same standard mouse diet and received tap water ad libitum. There were 10 mice in each group that were born in these cages. When the mice were 5–6 weeks old, both the mother and the siblings were removed from the cages, with the male experimental mice remaining in the same cage. The mice grew up and were housed for 6 months in the assigned type of cage in which they were born. For all the three groups, the 6 months period started within the same week and ended within the same week to exclude all time dependent effects. In the cages.

- 2 mice per IVC housed together
- 2 mice per CC500 housed together, and
- 4 mice per CC800 housed together.

The area per mouse was therefore almost the same in each cage. In the second and the third month, slightly aggressive behavior was observed in CC800 cages where 4 mice were housed together, but there was no tail or body injury so the animals were not separated. To enrich the cage environment, a red, transparent plexiglass house was placed in the cages. In addition, hard paper cylinders and paper tissues were provided in the cages. For the CC800 group, 3 cages each consisted of 4 mice were formed. For 6 months they stayed together until decapitation. However, in order to achieve *n* = 10 in the CC800 group (as in the other two groups), only 2 mice from the 3rd CC800 cage were included in the study to accommodate sample size for groups. In other words, only 2 mice were included in the behavioral tests. At the end of 6 months, the body weights of the animals were measured.

### Behavioral tests

Following 180 days, when the animals were 6-month-old, the hang wire test and the adhesive removal test were performed to test cortical sensory-motor functions. The next day, the forced swim test was performed to assess the distress and depression like behavior. These experiments were carried out in the animals' own habitats. A regular pattern was avoided when selecting the cages for the experiments. The tests were performed every day between 9:00 and 12:00 in the morning. All behavioral tests were recorded with a camera and a computer. All scoring and analysis were done and confirmed by offline evaluation of the video recordings.

### Hang wire test

Hang wire test was used to evaluate the grip strength, balance and endurance of mice [16, 17]. As detailed in our previous study [18], the mouse was placed on a stretched wire with a thickness of 0.2 cm. The mouse had to support its body weight with its forelimbs. A cushion pillow was placed 35 cm below the wire to prevent the mice from dangerous falls. Mice used their forelimbs to hang on the wire. The time until the mouse fell was recorded. If the mouse didn’t fall within 120 s, it was removed by the experimenter. The protocol for each mouse consisted of two repetitions, each of which also consisted of 2 trials (4 trials in total). There were 1 h intervals between two repeats and 2–3 min break between two trials. The average values of 4 trials were calculated for each animal and used for statistical analysis. In the hang wire test, holding time of the mice to wire was measured in seconds. (See the pictures and movie of the hang wire test in Additional file 1: Fig S1 and Additional file 2: Movie 1).

### Adhesive removal test

Adhesive removal test, was performed to assess sensorimotor deficits in mice. The healthy mouse feels the presence of a foreign material in its paw and the latency to remove the adhesive is measured. The time of the first touch to the adhesive and time of removal of the adhesive reflect sensorimotor performance [19, 20]. As previously described [18], a 3 × 3 mm square, red adhesive tape strips were stuck to the front paws of the mice. The time to touch one of the tapes with their nose or mouth and the time to remove each tape were used for statistical comparisons. The tests were recorded with a maximum test time of 3 min. The test for each mouse was repeated for three times and there were 2–3 min intervals between repetitions. In adhesive removal test, the performances were given in seconds. (See the pictures and movie of the adhesive removal test in Additional file 3: Fig S2 and Additional file 4: Movie 2).

### Forced swim test

Mice were placed in transparent beakers filled with water so deep that they could not escape. Their mobility behavior was measured when they tried to escape. Each mouse spent 6 min in the beaker; the immobility time was measured in the last 4 min. During the experiment, the temperature of the water was kept at 23–25 ℃ [21]. Since this test depends on a simple paradigm and requires a minimum number of equipment, it is more reliable. Successful implementation requires minimizing stress in mice and adhering to the details of the procedure [22]. At first, the mouse shows powerful activity as it tries to escape. Then it stops and displays a characteristic immobility in which it moves only to keep its head above the water. This physical inactivity is thought to be an indicator of behavioral hopelessness and depressive like behavior. Observers measure the time between placing the animal in the beaker and the onset of immobility. In rodent models of depression, the time spent trying to escape decreases over time [23]. (See a picture and a movie of the forced swim test in Additional file 5: Fig S3 and Additional file 6: Movie 3).

### Biochemical assays

One day after the last behavioral test, under isoflurane anesthesia, the mice were rapidly decapitated by using a manual guillotine (the 2 mice used to adjust the number in the CC800 were not included to the behavioral tests and not decapitated). Trunk blood was collected into tubes. The brains were rapidly removed. The procedures for blood and brain conducted at the same time by two researchers independently. Blood samples were collected to serum separation tubes and centrifuged at 1000xg for 20 min. Brain tissues were dissected with scalpel and forceps on ice-cold petri dish on filter paper soaked with saline solution. First, olfactory bulbs and cerebellum were removed. For frontal cortex, frontal pole (3–4 mm) was cut and subcortical regions were removed. Then, from occipital side, by visual cortex and brain stem pushed away and hippocampi were harvested. Finally, the sensory and motor areas cut out avoiding the visual cortex and subcortical tissues. The brain and serum samples were stored at − 80 °C until the biochemical analysis were done. Serum corticosterone levels were measured from blood serum samples by using the commercial ELISA kit (FineTest, Wuhan China; Cat Nr: EM0946). The basis of the kit was based on the competitive-ELISA detection method. After the procedure steps followed in line with the manufacturer's recommendations, absorbance was detected at 450 nm wavelength in the micro plate reader (VERSA Max Moleculer Devices). The results were calculated with the aid of a standard graph and expressed as ng/mL.

Rest of the biochemical measurements were performed for three different brain tissues: Frontal cortex, motor-sensory cortical region and hippocampus tissues. Approximately 25 mg of each tissue sample were taken into an eppendorf and 1 mL phosphate buffer was added (PBS, pH:7.4). The brain tissue was then homogenized by an ultrasonic homogenizer (SONICS Vibra Cell VCX 500, Connecticut USA). The homogenates were then centrifuged at 1800xg for 15 min. Supernatants were separated for measurement of biochemical parameters. Protein determination in supernatants was performed using BCA protein assay kit (PierceTm Thermo Scientific, USA).

Tissue malondaldehyde (MDA) measurement was performed based on the method developed by Mihara and Uchiyama [24]. The method is based on measuring the absorbance at 532 nm of the color formed by MDA with thiobarbituric acid in an acidic medium. Results were expressed as nmol/g protein.

Total Antioxidant Status (TAS) and Total Oxidant Status (TOS) determinations were made using colorimetric commercial kits in accordance with the manufacturer's recommendations (Rel Assay Diagnostics, Gaziantep, Turkey). Results for TOS were given as μmol H_2_O_2_ Equivalent/g protein, while TAS values were expressed as mmoL trolox equivalent/g protein.

### Statistical analysis

Data were presented as means ± SEM. Normality tests were done with Shapiro–Wilk test. All data were normally distributed so significant differences between groups were analyzed by one-way ANOVA using GraphPad Prism software. For multiple comparisons Tukey’s post hoc test was used. *p* < 0.05 was accepted as significant.

## Results

### Mice in the CC800 had higher body weights

**Fig. 2:**
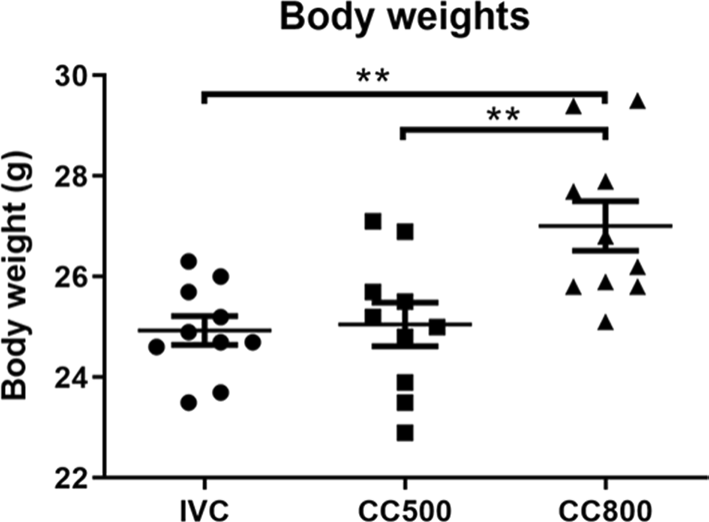
Effects of housing type on body weights. Mice lived in CC800 cages significantly heavier than the other groups of mice. (** *p* < 0.01, *n* = 10 for each group)

At the end of 6 months, the body weights were measured and it was found that there was a significant difference in body weights between animals housed in different cages (F_2,27_ = 8.033; *p* = 0.0018). The mice reared in CC800 (27.01 ± 0.49 g) were significantly heavier than those reared in CC500 (25.05 ± 0.43 g; *p* = 0.0063) and IVC (24.93 ± 0.29 g; *p* = 0.0038, One way ANOVA Tukey test) (Fig. 2).

### Performances in hang wire test were similar among groups

**Fig. 3:**
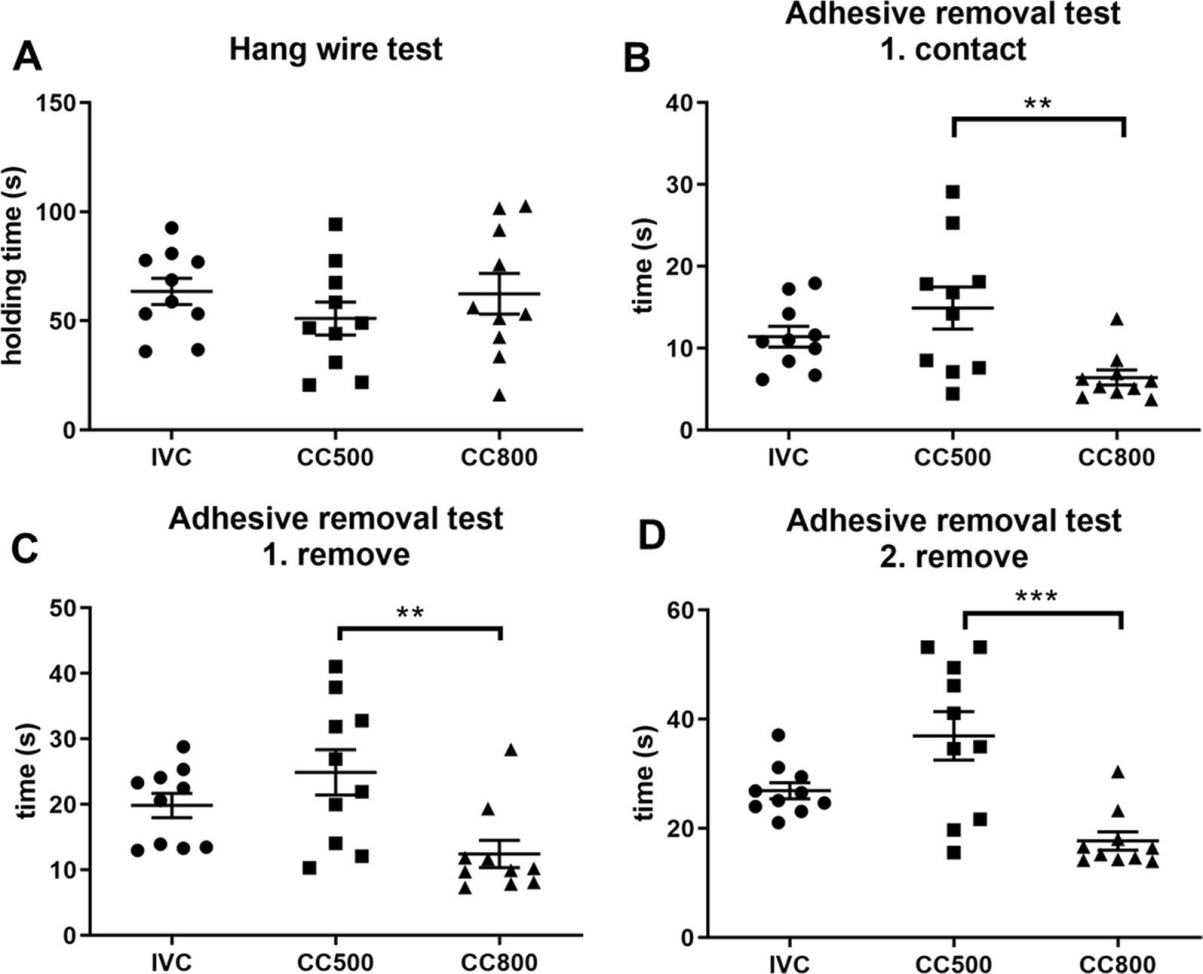
Sensory-motor performances. **A** Hang wire test showed no significant difference between the groups. **B** The time to first contact, **C** The removal of the first adhesive time and **D** the removal of the second adhesive were significantly longer in CC500 group. (**p < 0.01; ***p < 0.001, *n* = 10 for each group)

There was no significant difference between the groups in wire holding times (F_2,27_ = 0.782; *p* = 0.47). Figure 3A shows the average holding times during hang wire test.

### Mice in the CC800 had better performance in the adhesive removal test

The contact time to the tape and first and second removal times were measured in seconds (Fig. 3B). One way ANOVA test of contact time revealed a significant effect between groups (F_2,27_ = 6.01; *p* = 0.007). The contact time to the tape in CC500 group (14.91 ± 2.58 s) was longer than CC800 group (6.42 ± 0.92 s; *p* = 0.0051) but it is not significantly different from IVC (11.42 ± 1.27 s) group. Moreover, the first (F_2,27_ = 5.97; *p* = 0.0071) and the second (F_2,27_) = 11.24; *p* = 0.0003) removal times of the tape were significantly different between groups. First removal times of the CC500 (24.90 ± 3.45 s) were significantly longer than CC800 group (12.44 ± 2.08 s; *p* = 0.0053) but not IVC (19.83 ± 1.87 s) group (Fig. 3C). The second trial, mice in CC500 group (36.98 ± 4.46 s) removed the tape significantly later than CC800 (17.71 ± 1.66 s; *p* = 0.0003) group but the difference between CC500 and IVC (26.92 ± 1.47 s) groups was not important (Fig. 3D).

### The results of the forced swim test were not altered

**Fig. 4:**
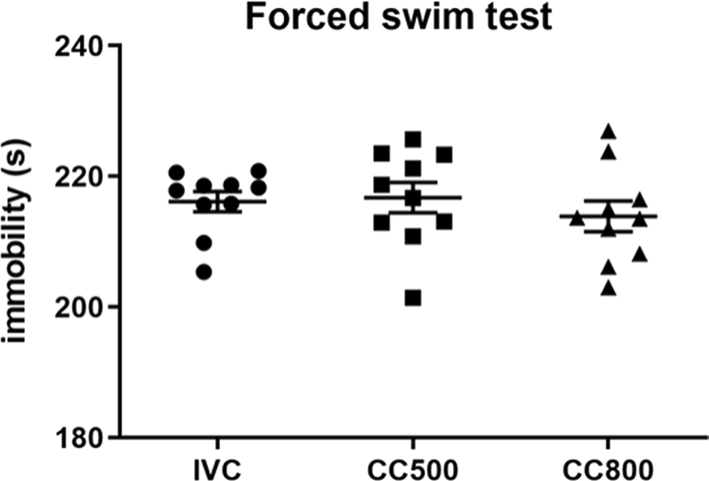
The results of forced swim test. Housing in different cage systems did not have a significant effect on forced swim test. The durations of immobility were similar in all groups (*n* = 10)

There was no significant effect of different caging on immobility and mobility times (F_2, 27_ = 0.51; *p* = 0.6) in the forced swim test (Fig. 4).

### Corticosterone levels were elevated in the CC800 group

**Fig. 5:**
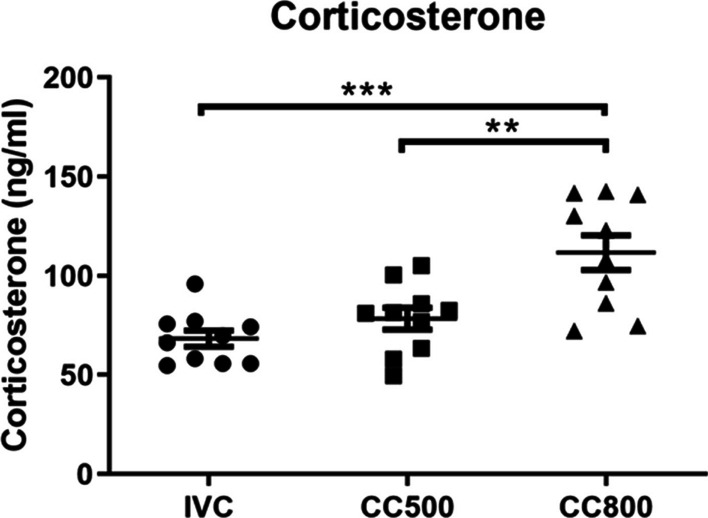
Serum corticosterone levels. Mice in the CC800 group have higher corticosterone concentrations than the other groups. (**p < 0.01; ***p < 0.001, *n* = 10 for each group)

One way ANOVA revealed that there was a significant difference in serum corticosterone levels among housing groups (F_2,27_ = 12.27; *p* = 0.0002). The highest corticosterone level was in the CC800 groups (111.7 ± 8.82 ng/ml), and the lowest in IVC group (68.36 ± 4.13 ng/ml). Corticosterone level in the CC500 group was 78.45 ± 5.54 ng/ml. Tukey post hoc test showed that CC800 group’s corticosterone level was significantly higher compared to the IVC group (*p* = 0.0002) and CC500 group (*p* = 0.0032) (Fig. 5).

### Malondialdehyde (MDA) was higher in the CC500 group

**Fig. 6:**
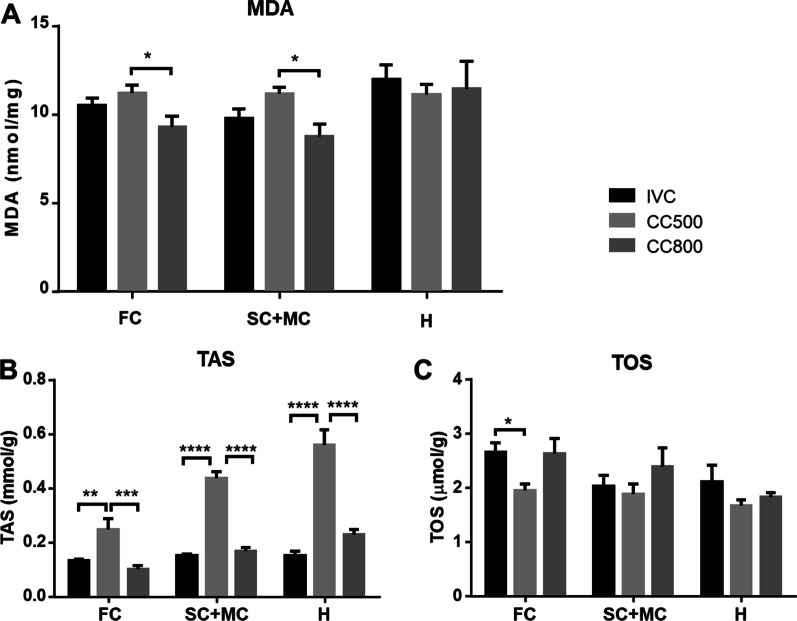
Oxidative stress parameters for three regions in the brain. The mean values for two cortical areas, frontal cortex (FC) and sensory-motor areas (SC + MC) as well as hippocampus tissues were summarized. **A** Malondialdehyde (MDA), **B** Total antioxidant status (TAS) and **C** Total oxidant status (TOS) of the groups. (*p < 0.05; **p < 0.01; ***p < 0.001, ****p < 0.0001, *n* = 10 for each group)

The MDA values of the frontal cortex tissue differed significantly among groups (F_2,27_ = 3.870; *p* = 0.0309). MDA levels in the frontal cortex were 10.48 ± 0.37 nmol/mg in IVC, 11.35 ± 0.4 nmol/mg in CC500 and 9.11 ± 0.72 nmol/mg in CC800. There was a significant difference between CC500 and CC800 (*p* = 0.0145). One way ANOVA revealed a significant difference in MDA levels of sensory-motor cortex (F_2,27_ = 5.58; *p* = 0.0094) between the groups. The MDA concentrations in the sensory-motor cortex were 9.55 ± 0.43 nmol/mg, 11.26 ± 0.38 nmol/mg and 8.80 ± 0.72 nmol/mg in IVC, CC500 and CC800, respectively. The MDA levels in sensory-motor cortex of CC500 group was higher than those of the CC800 group (*p* = 0.0082). The MDA levels analyzed from the hippocampal tissues were not significantly different between groups (F_2,27_ = 0.154; *p* = 0.85) (Fig. 6A).

### Total antioxidant status (TAS) levels were elevated in the CC500 group

The TAS values of all brain areas were significantly different among groups. One way ANOVA showed significance in the frontal cortex (F_2,27_ = 11.31; *p* = 0.0003), in the sensory-motor cortex region (F_2,27_ = 128.1; *p* < 0.0001) and in the hippocampus (F_2,27_ = 49.37; *p* < 0.0001). The TAS levels of CC500 group were significantly higher than those of the IVC group (*p* = 0.004 for frontal cortex, *p* < 0.0001 for sensory-motor cortex and p < 0.0001 for hippocampus) and CC800 group (*p* = 0.0003 for frontal, *p* < 0.0001 for cortical and *p* < 0.0001 for hippocampus) (Fig. 6B).

### Total oxidant status (TOS) levels were elevated in the CC500 group

The TOS levels of the frontal cortex tissue were significantly different among groups (F_2,27_ = 4.394; *p* = 0.022). The TOS levels of IVC group were higher than CC500 group (p = 0.037). Cortex (F_2,27_ = 0.668; *p* = 0.52) and hippocampus (F_2,27_ = 0.922; *p* = 0.41) TOS levels were not significantly different among groups (Fig. 6C).

## Discussion

It is discussed that the housing conditions and cage types used to house laboratory animals have a decisive influence on the physiology of the animals. In particular, the effects of IVC on animal behavior and stress have been discussed. In this study, with three groups of mice living in assigned cage from birth to adulthood, we showed that;

i. IVC cages did not lead to increased corticosterone levels, depressive behavior, abnormal body weights, impaired sensory-motor functions or elevated oxidative stress in different brain regions.
ii. The number of mice living in one cage appears to be a more severe factor for corticosterone levels, behavior and oxidative stress in the brain of laboratory mice.

During the 6 months, no fight or injury was observed in any of the cages. At the end of 6 months, the body weights of mice in CC800 group were higher than in the IVC group and the CC500 group. It is important to note that these mice were not transferred to a new cage i.e. they were born in that same cage. From birth to 6 months of age, the body weights of IVC group were not higher than that of the classical cages. There are contradictory results reported by others. The body weights of IVC group were higher than those of the classical cage group [25]. In another study, no difference in weight gain was found between IVC and open top classical cages [11, 26]. Moreover, significant differences in weight gain have been shown between different IVC systems [27] or after transferring the animal from classical cage to the IVC [12]. In our study, we did not measure the food intake. However, some studies have found lower food consumption in IVC cages [25, 26]. They suggest that the high level of air ventilation noise in IVC systems may minimize food and water intake [28]. Life from birth to adulthood in IVC cages did not lead to overweight or an obese phenotype in our study. In contrast, mice lived in the CC800 cages (larger cages but 4 mice per cage), had a significantly heavier body weight.

In the literature, there are studies examined various behavioral correlates of housing type. We have investigated the effects of different housing conditions on sensory-motor performances. We conducted hang wire and adhesive removal tests that are used to assess sensory motor performance. To the best of our knowledge, this is the first study to show that IVC cages have no negative effect on the sensory-motor performances. In the hang wire test, the CC500 group showed the worst performance. The performances of IVC and CC800 groups were similar and better than that of the CC500 group, although statistically not significant. In adhesive removal test, an increase in the time to first contact, removal of the first tape and removal of the second tape was observed in the mice of the CC500 group. They showed relatively poorer performance in both somatosensory and motor function. The CC800 group showed the best performance in adhesive removal test.

The performances in the forced swim test, which indicates the animals’ level of depression, were also compared. There were no significant differences between the immobility times, reflecting the depression like behavior of the mice. Housing in IVC cages did not lead to increased depression in the mice. This result is consistent with previously published data in which the housing system had no effect on the forced swim test [11].

Recently, it was reported that caging system modulates the turnover rate of dopamine (DA) and serotonin (5-HT), but not absolute levels of neurotransmitters [12]. IVC groups have increased turnover rate of DA and 5HT. It is known that DA is an important neurotransmitter in motor coordination [29, 30], and together with 5-HT, DA plays a remarkable role in the control of behavior and emotions [31, 32]. The change in sensory motor behavior in our study may be related to the turnover rates of these neurotransmitters.

In this study, we found that serum corticosterone levels were significantly higher in the CC800 group than in both the IVC and CC500 groups. In contrast to our results, a previous study, showed that maternal serum corticosterone levels were increased in pregnant mice in IVC cages compared to open cages after maternal immune activation (MIA) [33]. Consistent with this study, David and coworkers [5] has also found an increased cold stress response in mice kept in IVC systems compared to classical open cages [5]. In contrast to these findings, another study reported that neither the number of mice in the cage nor the type of cage changed the concentration of fecal corticosterone metabolites [34]. Whereas in our study, the CC800 group has significantly higher corticosterone levels which may indicate that there were more stressful conditions were present in the large CC800 cages where four mice were housed together. Besides, the CC800 cages contained 4 mice and the area per mouse is nearly 50 cm^2^ smaller than the CC500 and IVC. Increased stress in CC800 cages could also be related to the smaller area per mouse. This could have increased the stress levels in this cage. These discrepancies between the different studies can be explained by the different nature of control and IVC systems, the transportation of the animals, the number of mice in the cages, etc. Interestingly, the corticosterone levels did not correlate with oxidative stress levels in the brain. In the CC500 group but not in the CC800 group, oxidative stress parameters, cortical MDA and TAS levels were significantly higher than in the other two cages. Besides, rodents housed in IVC cages increased c-Fos expression in the paraventricular nucleus of the hypothalamus [6], which is an indicator of activation of stress responses [7]. Our finding also showed that the CC800 group had highest corticosterone concentrations and was heavier than the other groups. We can suggest that increased corticosterone may have led to higher body weight in the CC800 group. The mechanistic link between stress hormones and weight gain has been extensively studied [35]. Following experimental stress protocols, both a decrease [36] and an increase [37] in body weights was observed in mice. After oral chronic corticosterone intake, the body weights of the mice were increased, which was attributed to increased serum leptin levels and insulin resistance [38]. Our results revealed that high number of mice in the cage without additional stress led to higher corticosterone levels and higher body weights.

Food consumption is an important parameter in studies on stress. One limitation of the present study is that we did not measure the food consumption. To obtain accurate results, metabolic cages are required, which again contradicts the basic idea of the study. The other option is to weigh the food daily. This approach does not provide accurate data as some of the food is wasted by spilling into the cage. We have also avoided such daily measurement to mimic routine application in a common animal facility. However, the inclusion of food consumption data would definitely be useful.

## Conclusions

The current study shows that different caging systems and social group sizes influence somatosensory-motor behavior, body weight, plasma corticosterone and brain MDA levels. Contrary to popular belief, mice raised in IVC cages were not exposed to more stress compared to mice in open cages as shown by plasma corticosterone levels. In fact, the depression level of the mice in IVC cage group did not was no different than the other two open cages indicated by forced swim test. Moreover, the mice kept in IVC cage represent better performance in sensory motor behavioral tests. There are conflicting results reported in different studies on the stressful effects of IVC housing. Our results suggest that, IVC caging does not induce any additional stress in mice. The reported effects could be related to the transport of animals from classical cages to IVC rather than the direct effects of IVC systems. Furthermore, the present study confirms that IVC cage systems are safe alternatives for housing animals. For better comparability, reliability and reproducibility of the experimental results, the housing conditions of the mice should be clearly explained and these conditions should not be changed during the experiment.

### Supplementary Information

**Additional file 1 Fig S1.** Two photos from hang wire test.

**Additional file 2 Movie 1.** A movie from hang wire test. Below a hollow cushion is visible to prevent the harmful impact if mice fall off the wire.

**Additional file 3 Fig S2.** Two photos form adhesive removal test.

**Additional file 4 Movie 2. **A movie from adhesive removal test. In the video, mouse first touches to the adhesive tape and then removes the first and the second tape from its paws. The red tape is visible in the paw of a mouse.

**Additional file 5 Fig S3. **A photo from forced swim test.

**Additional file 6 Movie 3.** A movie from forced swim test. Two containers used for the swim tests. A white object (styrofoam) is used to visually isolate the two containers.

## Data Availability

The data of behavioral experiments, including the video recordings, and biochemical assays that support the findings of the present study are available from the corresponding author, [S.A.A.], upon reasonable request.

## Abbreviations

IVC: Individually ventilated cages
CC: Classical cage
MDA: Malondialdehyde
TAS: Total antioxidant status
TOS: Total oxidant status
5HT: Serotonin
DA: Dopamine
MIA: Maternal immune activation
