# Supplementary figures and images for: Effects of housing conditions on stress, depressive like behavior and sensory-motor performances of C57BL/6 mice
Source: Lab Anim Res. 2024 Feb 18;40:6. doi: 10.1186/s42826-024-00193-8 (PMC10874523; doi:10.1186/s42826-024-00193-8)

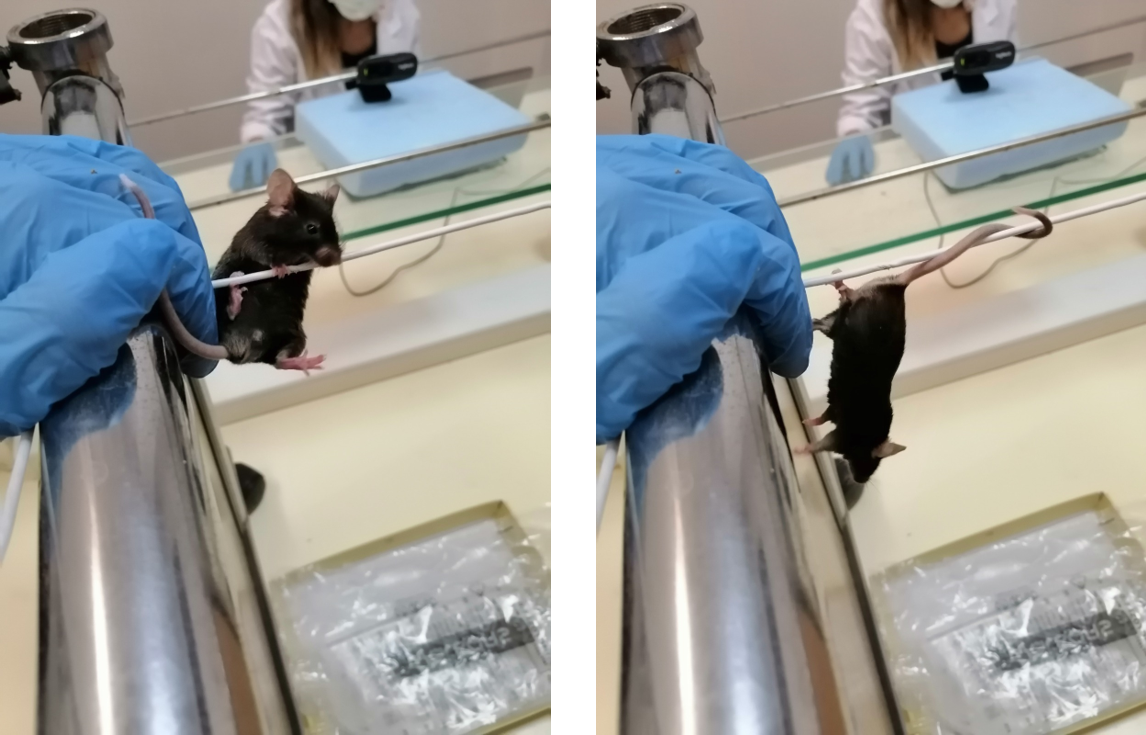

Supplement: Supplementary file 1 — Additional file 1 Fig S1. Two photos from hang wire test. [file 42826_2024_193_MOESM1_ESM.tif]

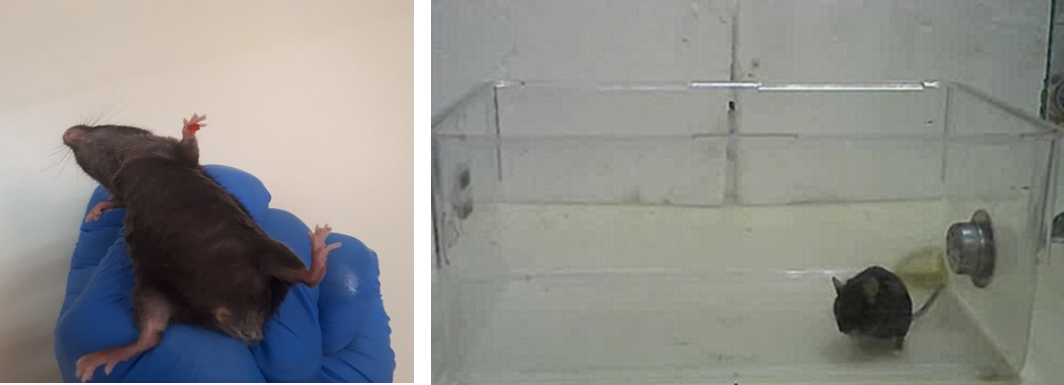

Supplement: Supplementary file 3 — Additional file 3 Fig S2. Two photos form adhesive removal test. [file 42826_2024_193_MOESM3_ESM.tif]

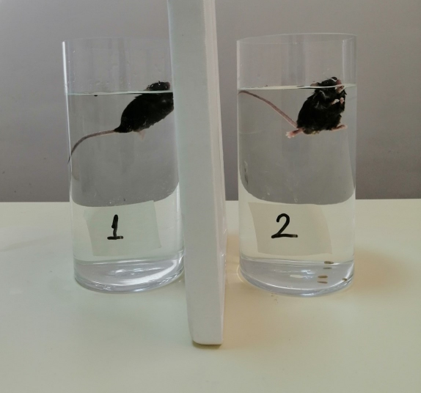

Supplement: Supplementary file 5 — Additional file 5 Fig S3. A photo from forced swim test. [file 42826_2024_193_MOESM5_ESM.tif]
